# Supplementary material for: The Bioactive Phenolic Agents Diaryl Ether CVB2-61 and Diarylheptanoid CVB4-57 as Connexin Hemichannel Blockers
Source: Pharmaceuticals (Basel). 2022 Sep 21;15(10):1173. doi: 10.3390/ph15101173 (PMC9611528; doi:10.3390/ph15101173)
Supplement: Supplementary file 1 [file pharmaceuticals-15-01173-s001.zip › pharmaceuticals-1873283-supplementary.pdf]

Supplementary Materials: The following supporting information can be downloaded at: www.mdpi.com/xxx/s1. Figure S1: Expression of the connexin variants in the N2A and HeLa cells. Figure S2a: Dye uptake in N2A and HeLa cells expressing only GFP and in non-transfected cells. Figure S2b: Dye uptake in HeLa cells expressing connexins. Figure S3: Representative experiments showing inhibition of the dye uptake by CVB2-61 or CVB4-57 in N2A cells expressing Cx26 and Cx46. Figure S4: Representative experiments showing dye uptake and its inhibition by CVB2-61 or CVB4-57 in Calu-3 cells.

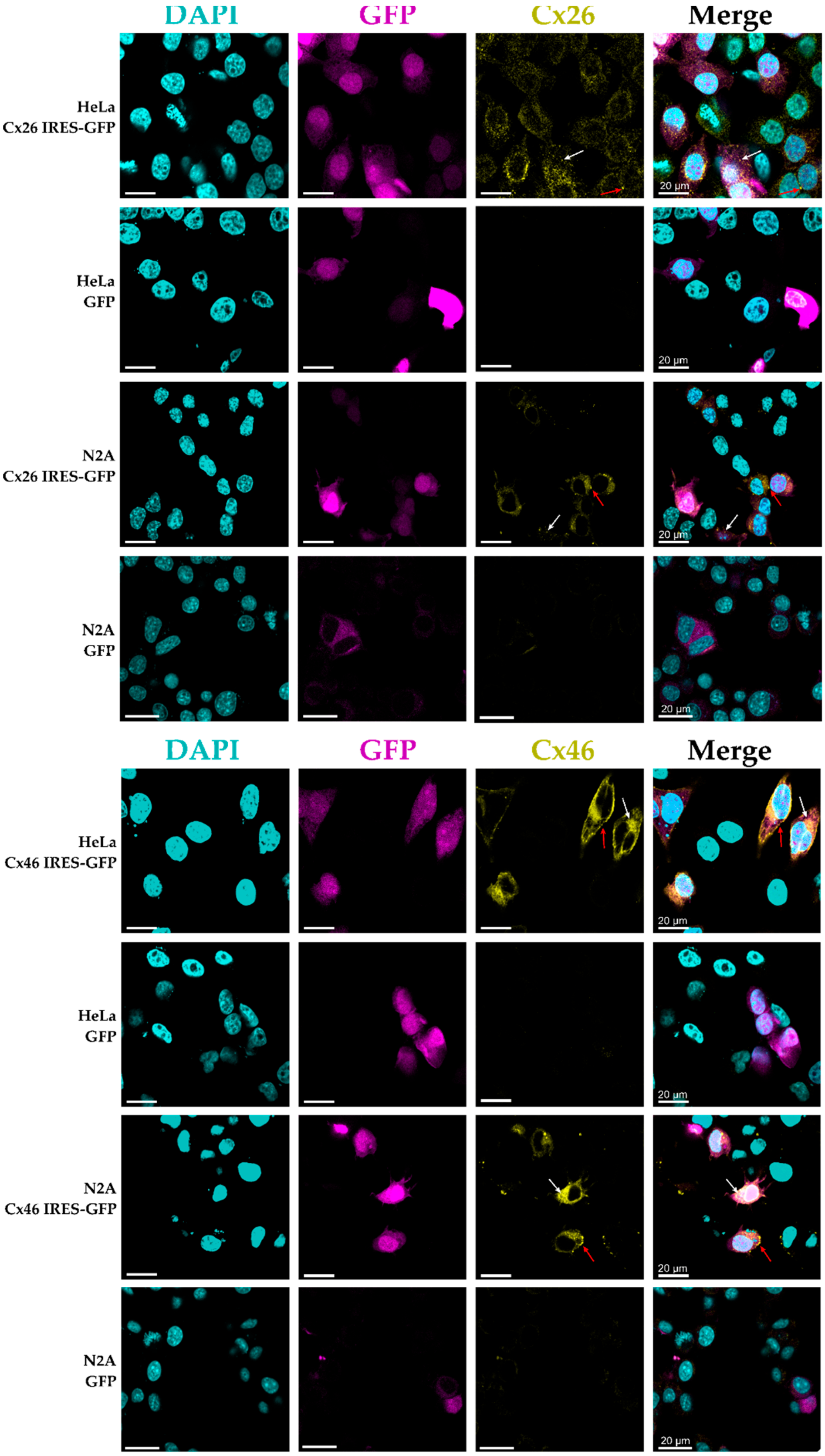

Figure S1. Expression of the connexin variants in the N2A cells and HeLa cells. Transfected cells were recognised by the expression of GFP (magenta) in the cell cytoplasm. For orientation the cell nuclei (cyan) were stained with DAPI. The Cx (yellow) were stained using respective antibodies. Cxs were mostly found at the cell border (red arrows). Cytoplasmic localisation (white arrows) in structures, probably vesicles, endoplasmic reticulum, or Golgi apparatus, was also observed.

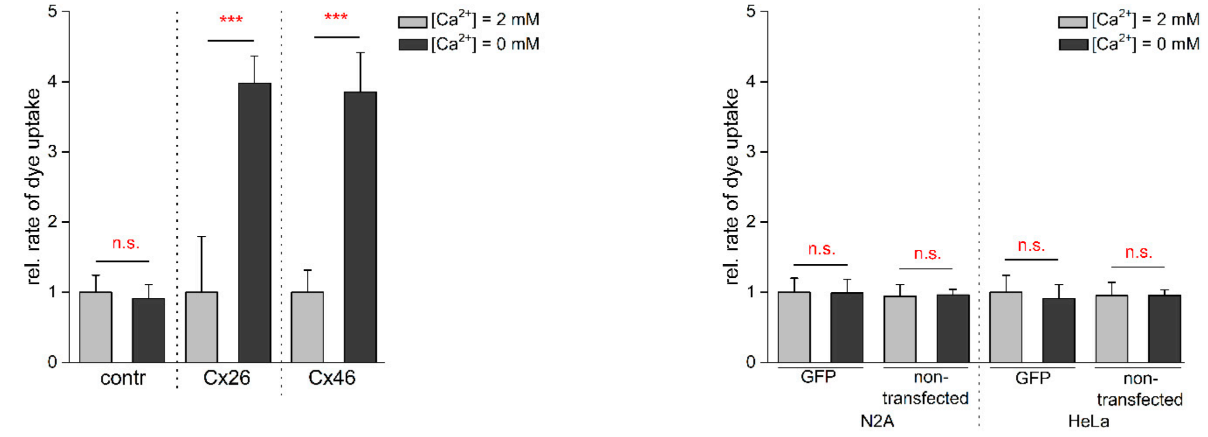

(a) **(b)**

Figure S2. (**a**) Expression of GFP did not change dye uptake. Quantification of the Etd uptake in non-transfected and GFP-containing vector-transfected N2A and HeLa cells. Independently of the transfection, the removal of external Ca^{2+} did not markedly change the rate of dye uptake. The results are given as mean ± SEM of at least three respective and independent transfections. One-way ANOVA was applied for statistical significance in relation to the representative control. (**b**) Dye uptake in HeLa cells expressing connexins. Quantification of the rate of Etd uptake in the HeLa cells in the presence of and after removal of external Ca^{2+}. The rate of dye uptake was enhanced in HeLa cells transfected with Cx IRES-GFP variants as compared to cells only expressing GFP (contr). The results are given as average ± SEM of at least three respective experiments. One-way ANOVA was applied for statistical significance in relation to the control (***: $p < 0.001$).

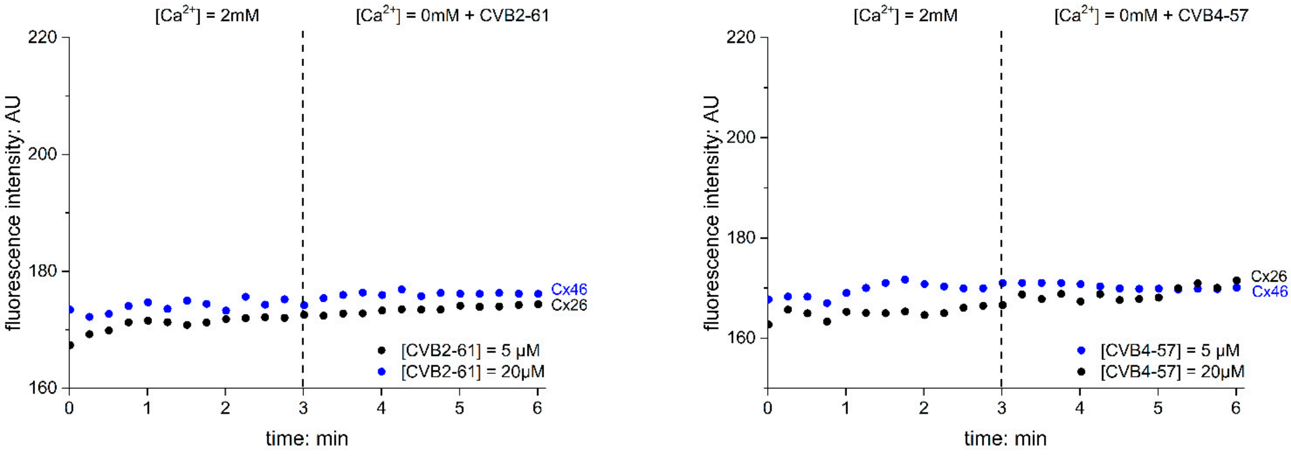

(a) **(b)**

Figure S3. Inhibition of dye uptake by the agents CVB2-61 or CVB4-57. Representative experiments showing the time course of the fluorescence intensity of Etd in N2A cells transfected with hCx26IRES-GFP (Cx26) vector or Cx46IRES-GFP vector (Cx46) in the presence of and after removal of external Ca^{2+} in the presence of CVB2-61 or CVB4-57 at the indicated concentrations. (**a**) [CVB2-61] = 5 μM, [CVB2-61] = 20μM; (**b**) [CVB4-57] = 5 μM, [CVB4-57] = 20μM.

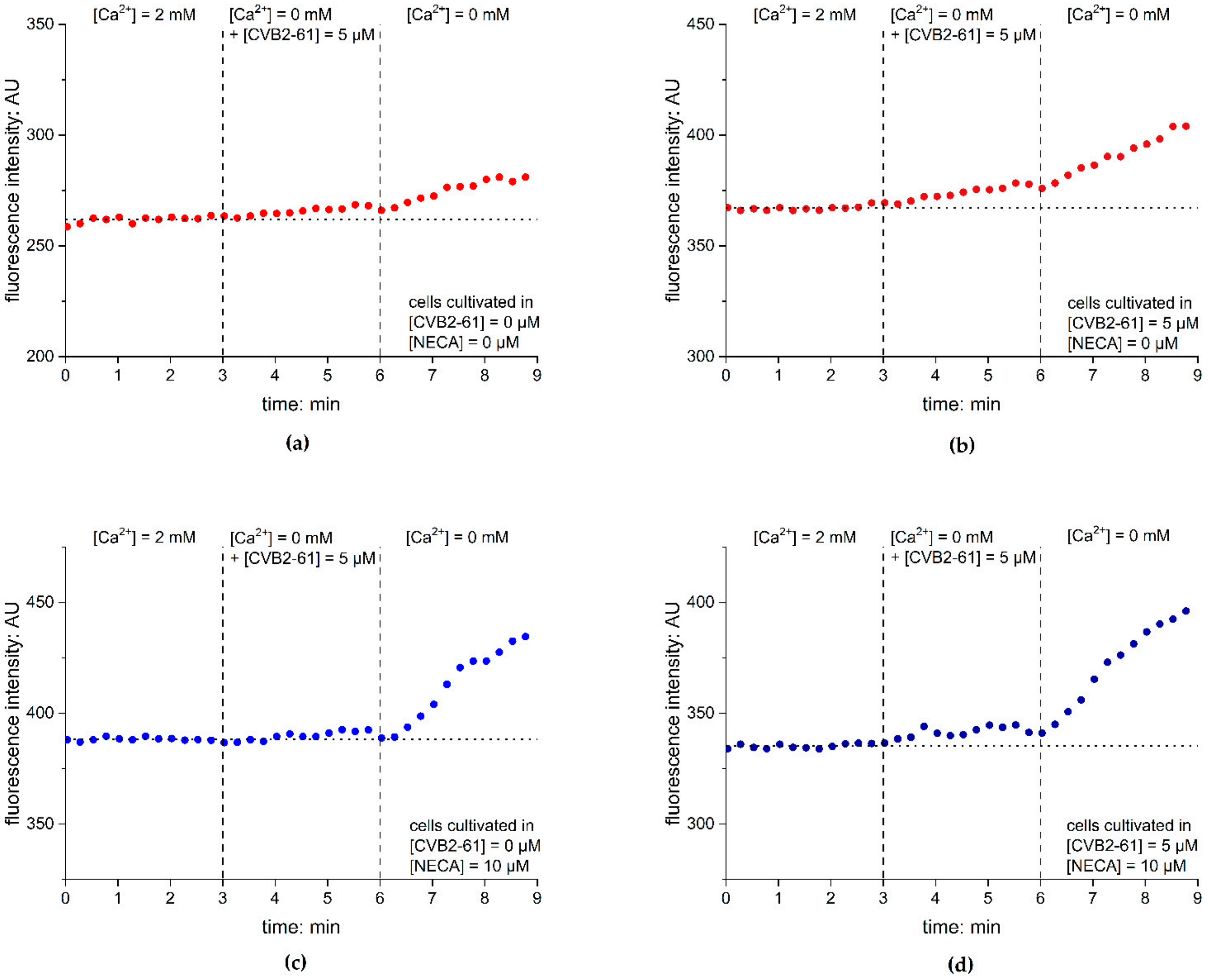

Figure S4. CVB2-61 and CVB4-57 inhibited the dye uptake in Calu-3 cells. Representative experiments showing the time course of the fluorescence intensity of Etd in Calu-3 cells treated as indicated. (**a**) cells cultivated in[CVB2-61] = 0 µM [NECA] = 0 µM; (**b**) cells cultivated in[CVB2-61] = 5 µM[NECA] = 0 uM; (**c**) cells cultivated in[CVB2-61] = 0 µM[NECA] = 10 µM; (**d**) cells cultivated in[CVB2-61] = 5 µM[NECA] = 10 µM.
